# Health care providers attitude towards safe abortion care and its associated factors in Northwest, Ethiopia, 2021: a health facility-based cross-sectional study

**DOI:** 10.1186/s12978-024-01826-w

**Published:** 2024-06-08

**Authors:** Abebay Tadie Wubetu, Alemtsehay Mekonnen Munea, Wondu Feyisa Balcha, Fentahun Alemnew Chekole, Amanuel Tebabal Nega, Alemwork Abie Getu, Melash Belachew Asresie

**Affiliations:** 1 Felege Hiwot Comprehensive Specialized Hospital, P. Box: 079, Bahir Dar, Ethiopia; 2 Department of Reproductive Health and Population Studies, College of Medicine and Health Sciences, Bahir Dar University, P. Box: 079, Bahir Dar, Ethiopia; 3 Department of Midwifery, College of Medicine and Health Sciences, Bahir Dar University, P. Box: 079, Bahir Dar, Ethiopia

**Keywords:** Abortion, Attitude, Health care provider, Favorable, Knowledge, Safe abortion

## Abstract

**Background:**

A negative attitude towards abortion among health care providers providing abortion services could be an obstacle even under a law, which permits abortion on request. Healthcare providers are expected to perform and be change agents of abortion services. However, little information is known about the attitude toward safe abortion among healthcare providers in Ethiopia.

**Objective:**

This study aimed to assess health care provider’s attitudes towards safe abortion care and its associated factors at the public health facilities of Bahir Dar City, Northwest Ethiopia.

**Methods:**

A health facility-based cross-sectional study was employed from March 1 to 30/2021 among 416 health-care providers. The data were collected by computer-based generated simple random sampling technique, entered, coded, and cleaned using Epi data version 4.2 and analyzed using Statistical Package of Social Sciences version 25.0. Bivariate and multivariable logistic regression analyses were employed to estimate the crude and adjusted odds ratio with a confidence interval of 95% and a P-value of less than 0.05 considered statistically significant.

**Results:**

The response rate of the study was 99.3%, and 70.2% [95% CI: 65.6–74.6] of health-care providers had a favorable attitude towards safe abortion care. Multivariable analysis indicated that health care providers who are found in the age group of 25–29, 30–34, and ≥ 35 years [AOR = 3.34, 95% CI = 1.03–10.85], [AOR = 4.58, 95% CI = 1.33- 15.83] and [AOR = 5.30, 95% CI = 1.43–19.66] respectively, male health care providers [AOR = 3.20, 95% CI = 1.55–6.60], midwives [AOR = 6.50, 95% CI = 2.40–17.44], working at hospital [AOR = 4.77, 95% CI = 1.53–14.91], ever trained on safe abortion [AOR = 5.09, 95% CI = 2.29–11.32], practicing of an abortion procedure [AOR = 2.52, 95%, CI = 1.13–5.60], knowledge of abortion [AOR = 7.35, 95% CI = 3.23–16.71], awareness on revised abortion law [AOR = 6.44, 95% CI = 3.15–13.17] and need further legalization of abortion law [AOR = 11.78, 95% CI = 5.52–24.26] were associated with a favorable attitude towards safe abortion care.

**Conclusions:**

Healthcare providers who had a favorable attitude toward safe abortion care were relatively high compared to the previous studies. Age, sex, profession, workplace, training, knowledge, and practice-related factors were associated with a favorable attitude toward safe abortion. This study indicated that, a need for intervention to help improve the attitude of healthcare providers toward safe abortion care, especially for those working in the maternity care units.

**Supplementary Information:**

The online version contains supplementary material available at 10.1186/s12978-024-01826-w.

## Introduction

Abortion is defined as the termination of pregnancy by the removal or expulsion of the fetus or embryo from the uterus before viability [1, 2]. An abortion can be spontaneous abortion or induced [3], and induced abortion is further classified into, safe or unsafe, legal or illegal, surgical or medical [4]. Globally, three out of ten of all pregnancies end in induced abortion [5], and in Africa and Latin America, more than three-fourths of all abortions are considered unsafe [6]. Unsafe abortion is described by the World Health Organization (WHO) as a procedure for ending an unintended pregnancy, either by people without the requisite expertise in a setting that does not comply with minimum medical requirements, or both [7, 8].

Globally, unsafe abortions account for over 47,000 maternal deaths per year (13% of total maternal mortality) and contribute to significant morbidity among women, especially in low-income countries [9–11]. Sub-Saharan African countries account for 86% of the world's illegal abortion [7, 12]. In Ethiopia, 38% of illegal abortions take place every year [13, 14]. In 2005, Ethiopia approved a liberalized abortion law [15], and this law allowed women to procure safe and legal abortion under certain conditions; these conditions included the following: if the pregnancy was due to abuse if there was physical or mental disability, if it put women at risk of physical health or life, or if the woman was younger than 18 years of age and unprepared to give birth [16].

To reduce unsafe abortion and its harmful complications article 551 of the penal code of the Federal Democratic Republic of Ethiopia allows termination of pregnancy under some conditions [5]. In June 2014, the Federal Ministry of Health also revised the technical and procedural guidelines for safe abortion facilities to assess the standard of care and also permitted the first-trimester pregnancy-safe abortion care can be given at the health center level as part of task sharing and task-shifting [17]. In the provision of abortion services, healthcare providers have an important role. However, the lack of healthcare providers in low-income countries is still critical and compounded by some healthcare providers' refusal to provide abortion services and this is again exacerbated by religious and cultural factors [18, 19].

Safe abortion is an important component of reproductive health care, providing a variety of medical and related health services, including therapy, contraception, and, where necessary, referrals to other reproductive health services [20]. Health-care providers' attitudes have potential consequences for women already with scarce access to safe abortion services [21]. Health-care providers are responsible for the provision of comprehensive abortion care services and are authorized to perform abortion procedures on women whose medical conditions warrant the immediate termination of pregnancy, which is an integral component of sexual and reproductive health [22, 23].

Health-care providers play a key role in the identification and treatment of complications, and in minimizing the burden of induced abortion, however, the attitude of healthcare providers is one of the key obstacles preventing women from obtaining safe abortion services [24, 25]. A study in southern Uganda shows that positive attitudes and behavior of healthcare professionals regarding safe abortion are contributing factors both for themselves and for the community [26]. Another study conducted in Nigeria shows nurses' attitudes are crucial in gaining and promoting patients' uptake of care and help to improve women's confidence [27]. Health-care providers with negative attitudes toward safe abortion may cause women with unplanned pregnancies to endanger their lives and pursue illegal abortions by untrained health workers [28, 29].

According to the study conducted in different parts of Ethiopia, the attitude of healthcare providers toward safe abortion ranged from 48.1% to 95% [30, 31]. Different factors affect the attitude of health care providers toward safe abortion care like age, religion, work experience, profession, awareness of the revised abortion law, knowledge of abortion, training on abortion, practicing of abortion, unpreparedness, and availability of service in the health facility and working facility [26, 30–34]. However, there is little information on attitude and its associated factors of safe abortion among health care providers in the study area. Therefore, this study aimed to assess the health care provider’s attitude toward safe abortion care and its associated factors at the public health facilities of Bahir Dar City, Northwest Ethiopia.

## Methods and materials

### Study design and period

A health facility-based cross-sectional study design was employed for this study from March, 1 to 30/2021.

### Study area

The study was conducted in the public health facilities of Bahir Dar city. Bahir Dar City is the capital city of the Amhara Region and is located approximately 565 km northwest of Addis Ababa, the capital city of Ethiopia. The estimated population of the city for the year 2020/21 is about 518, 193 of which 265,156 are females [35]. In the city, there are 3 public hospitals and six public health centers, 4 private general hospitals, 34 private medium clinics, 6 private lower clinics, and 3 Nongovernmental Organization clinics. All the public health facilities provide comprehensive abortion care services and the services are free for all women. There are 1757 health providers in the three public hospitals and six health centers of the city. Of these, 973 were female health workers.

### Source population

All health care providers who were working at the public health facilities of Bahir Dar city.

### Study population

Randomly selected healthcare providers who are working at the public health facilities of Bahir Dar City during the data collection period.

### Inclusion and exclusion criteria

Health professionals who are working in clinical departments such as general nurses, midwives, health officers, integrated emergency surgery officers, general practitioners, and specialists who were present during data collection time were included, while health care providers like pharmacists, laboratory technicians, radiology, environmental health, and free service health care providers were excluded.

### Sample size determination

The sample size was calculated using a single population proportion formula by considering the following assumptions: the proportion of health care providers who had a good attitude towards safe abortion care taken from the previous study 56.7% [36], Zα/2 = critical value for normal distribution at 95% confidence level, which is equal to 1.96 (Z value of alpha = 0.05) or 5% level of significance (α = 0.05) and a 5% margin of error (ω = 0.05). Then the sample size was calculated as follows (*n* = (Z/2)^2^p (1-p)/d2), (*n* = (1.96)2 0.567 (1–0.567)/0.05^2^) = 377. The sample size was adjusted by adding a 10% non-response rate and the final sample size was 377*10 = 416 health care providers.

### Sampling procedure and techniques

In Bahir Dar city, there are three public hospitals and six public health centers and all of these public health facilities of Bahir Dar city were included in this study. The total sample size was proportionally allocated for each public health facility of the city based on their total number of health care providers. Before data collection, the census was conducted to identify healthcare providers in each public health facility.

After the census, the total number of healthcare providers in the public health facility of the city was 1,758. The numbers of health care providers in Felege Hiwot Comprehensive Specialized Hospital (*n* = 847), Tibebe Gion specialized hospital (*n* = 576), Addisalem Primary Hospital (*n *= 154), and the six public health centers, including Bahir Dar health center, Han health center, Dagimawi Minilik health center, Shimbit health center, Shumoabo health center and Abaymado health center (*n* = 181). The total sample size was proportionally allocated for each health facility, based on its population size. The total sample size after proportional allocation was 201 for Felege Hiwot Comprehensive Specialized Hospital, 136 for Tibebe Gion Specialized Hospital, 36 for Addisalem Primary Hospital, and 43 for the six public health centers of the city. The study participants were selected by a computer-based simple random sampling technique.

### Study variables

#### Dependent variable

The attitude of health care providers towards safe abortion care (favorable/ unfavorable).

##### Independent variables

Socio-demographic factors (age, sex, marital status, and religion), health facility-related factors (type of health facilities, profession, work experience, and work unit), knowledge of safe abortion and awareness of the revised law, and practice-related factors (having training and practicing abortion procedure).

#### Operational definitions

##### Attitude toward safe abortion care

Refer to the attitude of health care providers towards safe abortion care and assessed using 20 questions by assigning + 1 for strongly disagree, + 2 for disagree, + 3 for neutral, + 4 for agree, and + 5 for strongly agree. Health care provider was considered to have a favorable attitude toward safe abortion care if he/she correctly answered greater than or equal to the mean score of the total attitude towards safe abortion assessing questions and those who scored less than the mean score were considered as having an unfavorable attitude towards safe abortion care [36].

##### Knowledge

Refers to the knowledge of health care providers about abortion and it has been assessed using 48 composite variables by assigning + 1 for a correct answer and 0 for an incorrect answer. Health care provider was considered to have good knowledge of abortion if he/she correctly answered greater than or equal to the mean score of the total knowledge of abortion assessing questions and those who scored less than the mean score were considered as having poor knowledge of abortion [37].

Further revision of abortion law: legalization of abortion which is open or unrestricted law as compared to the current law [36].

##### Awareness

Refers to the awareness of the revised abortion law of the country and it has been assessed using 5 composite variables by assigning + 1 for a correct answer and 0 for an incorrect answer. Healthcare provider was considered to have a good awareness of the revised abortion law if he/she correctly answered greater than or equal to the mean score of the total awareness of the revised abortion law assessing questions and those who scored less than the mean score were considered as having of poor awareness of the revised abortion law of the country.

##### Health care provider

A health care provider is an individual health professional licensed to provide services to the patient which includes midwives, nurses, health officers, general practitioners, integrated emergency surgery officers, and specialists [30, 38].

### Data collection tools and procedures

A structured self-administered questionnaire was used to collect the data which were adapted from relevant works of literature and modified to the local context [26, 30, 32, 36]. The questionnaires were prepared in the English language. The questionnaire consisted of socio-demographic characteristics, source of information-related factors, health facility-related factors, attitude, knowledge, and practice-related factors questions. A Pre-tested structured interviewer-administered questionnaire was used for data collection purposes. The data were collected by six diploma midwives and supervised by two BSc midwives.

#### Data quality control

Data were collected by trained data collectors and pretesting of the instrument was done before the actual data collection. The questionnaire was pre-tested before the actual data collection period on 21 healthcare providers, or (5%) of the sample size at Dangila Primary Hospital, to assess the reliability, clarity, sequence, consistency, understandability, and the total time it takes to finish the questionnaire. Data collectors and supervisors were trained for two days by the investigator. After necessary modifications and correction was done to standardize and ensure its reliability and validity additional adjustments were made based on the results of the pre-test. The Cronbach alpha score for the pretest was 0.8. The completeness of the data was checked by data collectors during data collection and daily supervision was done for data completeness by supervisors.

#### Data processing and statistical analysis

The data were entered into Epi data 4.2, edited and cleaned for inconsistencies, missing values, and outliers, and then exported to SPSS version 25.0 for analysis. During analysis, all explanatory variables that have a significant association in bivariate analysis with a *P*-value < 0.25 were entered into a multivariable logistic regression model to get AOR and those variables with 95% CI and a *P*-value of < 0.05 were considered as statistically significant with a favorable attitude of health care provider towards safe abortion care. The multicollinearity test was done using the variance inflation factor and there was no collinearity between the independent variables. The model goodness of the test was checked by using Hosmer- Lemeshow goodness of the fit and its *P*-value was 0.215. Frequency tables, figures, and descriptive summaries were used to describe the study variables. A preprint version of the paper has previously been published, *Tadie A. *et al*.* 10.21203/rs.3.rs-2305231/v1 [39].

## Ethics declarations

### Ethics approval and consent to participate

Ethical clearance was obtained from the Institutional Review Board of Bahir Dar University, College of Medicine and Health Sciences. Further approval was also granted from Amhara public health institutions and respective health facilities. The purpose of the study was explained to each healthcare provider. At the time of data collection, written consent was obtained from each study participant. The study was conducted according to the recommendations of the Code of Ethics of the World Medical Association (Declaration of Helsinki). All respondents were assured that the data would not have any negative consequences on any aspect of their life.

## Results

### Socio-demographic characteristics of healthcare providers

A total of 413 healthcare providers participated in the study with a response rate of 99.3%. The mean age of the healthcare provider was 30.28 years. Of the total healthcare providers, 221 (53.5%) are female and 201 (48.7%) of the participants were nurses. Nearly half (*n* = 204, 49.4%) of the healthcare providers had 6–10 years of work experience (Table 1).

**Table 1:** Socio-demographic characteristics of health care providers at the public health facilities in Bahir Dar City, Northwest, Ethiopia, 2021, (*n* = 413)

Variables	No. (%)
Age of health care providers in years	
20–24	35 (8.5)
25–29	158 (38.3)
30–34	138 (33.4)
35–39	48 (11.6)
≥40	34 (8.2)
Sex of healthcare providers	
Female	221 (53.5)
Male	192 (46.5)
Religion	
Orthodox	343 (83.1)
Muslim	41 (9.9)
Protestant	23 (5.6)
Catholic	6 (1.4)
Marital status	
Married	281 (68.0)
Single	126 (30.5)
Others^a^	6 (1.5)
Profession	
Nurse	201 (48.7)
Midwives	111 (26.9)
Health officer	28 (6.7)
General practitioner	52 (12.6)
Specialists	19 (4.6)
Integrated emergency surgery officer	2 (0.5)
Working facility	
Health center	42 (10.2)
Hospital	371 (89.8)
Working unit	
Maternal and Child Heath	106 (25.7)
Outpatient department	95 (23.0)
Medical ward	54 (13.1)
Surgical ward	32 (7.7)
Pediatric ward	26 (6.3)
Gynecology ward	45 (10.9)
Others^b^	55 (13.3)
Work experience	
Less than 1 year	14 (3.4)
1–5 years	119 (28.8)
6–10 years	204 (49.4)
More than ten years	76 (18.4)

^a^Widowed and divorced, ^b^Emergency, Neonatal intensive care unit, Recovery, and Operation room

### Knowledge of health care providers on abortion

In this study, 354 (85.7%) of the healthcare providers had good knowledge of abortion and 380 (92.0%) of the participants knew the definition of abortion in the Ethiopian context. Nearly 79% responded that safe abortion is the termination of pregnancy before 12 weeks of gestational age and 365 (88.4%) responded that raped women should submit evidence of rape. About 95.0% of the participants responded that medical abortion is one of the abortion methods and 306 (74.1%) responded that equipped health facilities with trained staff are authorized to perform the procedure were the place for terminating a pregnancy as permitted by the revised abortion law of Ethiopia. More than three-fourths of the health care providers responded that manual vacuum evacuation, medication abortion, dilatation and evacuation, and 2nd-trimester abortion procedures have to be performed by a specialist. In our study, 376 (91.0%) of the participants responded that referral arrangement for social support and care is an integral part of overall abortion care and nearly 80.0% answered that health centers expected to give 1st-trimester safe abortion services. Over 92.0% of the participants responded that midwives can provide education on the legal provision of safe abortion services (Table 2).

**Table 2:** Knowledge measuring question on abortion among health care providers at the public health facilities in Bahir Dar City, Northwest, Ethiopia, 2021, (*n* = 413)

Variables	No. (%)
Abortion in Ethiopia's context	
Before 20 weeks of gestational age	33 (8.0)
Before 28 weeks of gestational age	380 (92.0)
Best time for safe termination of pregnancy	
After 12 weeks of gestational age	87 (21.1)
Before 12 weeks of gestational age	326 (78.9)
Raped women should submit evidence of rape	
Yes	365 (88.4)
No	46 (11.6)
Women who become pregnant by incest should submit evidence	
Yes	282 (68.3)
No	131 (31.7)
The health care provider has to be secure on informed consent using a standard consent form	
Yes	331 (80.1)
No	82 (19.9)
The health care provider has to be confidential, not disclose the information	
Yes	324 (78.5)
No	89 (21.5)
Abortion should be performed in equipped health facilities with trained staff who are authorized to perform the procedure	
Yes	306 (74.1)
No	107 (25.9)
Types of abortion methods (multiple responses were possible)	
Manual vacuum aspiration	304 (73.6)
Medical abortion	392 (94.9)
Dilatation and evacuation	153 (37.0)
Oxytocin induction	206 (49.9)
Components of safe abortion care (multiple responses were possible)	
Counselling	324 (78.5)
Provider partnership	148 (35.8)
Treatment of complications of unsafe abortion	361 (87.4)
Contraceptive and family planning service provision	289 (70.0)
Integration into reproductive health and other services	154 (37.3)
Manual vacuum aspiration performed by (multiple responses was possible)	
Specialist	374 (90.6)
Integrated emergency surgery officer	371 (85.5)
General practitioner	353 (87.4)
Health officer	269 (65.1)
Midwives	319 (77.2)
Nurses	251 (60.8)
Medication abortion performed by (multiple responses was possible)	
Specialist	380 (92.0)
Integrated emergency surgery officer	271 (65.6)
General practitioner	281 (68.1)
Health Officer	148 (35.8)
Midwives	254 (61.5)
Nurses	144 (34.9)
Dilatation and evacuation performed by (multiple responses were possible)	
Specialist	361 (87.4)
Integrated emergency surgery officer	186 (45.0)
General practitioner	53 (12.8)
Health Officer	30 (7.3)
Midwives	39 (9.4)
Nurses	29 (7.0)
2^nd^-trimester abortion procedures performed by (multiple responses were possible)	
Specialist	362 (87.7)
Integrated emergency surgery officer	329 (79.7)
General practitioner	326 (78.9)
Health Officer	211 (51.1)
Midwives	271 (65.6)
Nurses	146 (35.4)
The referral is an integral part of overall abortion care	
No	37 (9.0)
Yes	376 (91.0)
Health centers expected to give a 1st-trimester safe abortion	
No	84 (20.3)
Yes	329 (79.7)
Education on laws of abortion should be given by (multiple responses were possible)	
Specialists	361 (87.4)
Integrated emergency surgery officer	372 (90.1)
General practitioner	353 (85.5)
Health Officer	332 (86.0)
Midwives	355 (92.3)
Nurses	266 (64.4)
Knowledge of abortion	
Good knowledge	354 (85.7)
Poor knowledge	59 (14.3)

### Awareness of healthcare providers on the revised abortion law of Ethiopia

**Fig. 1:**
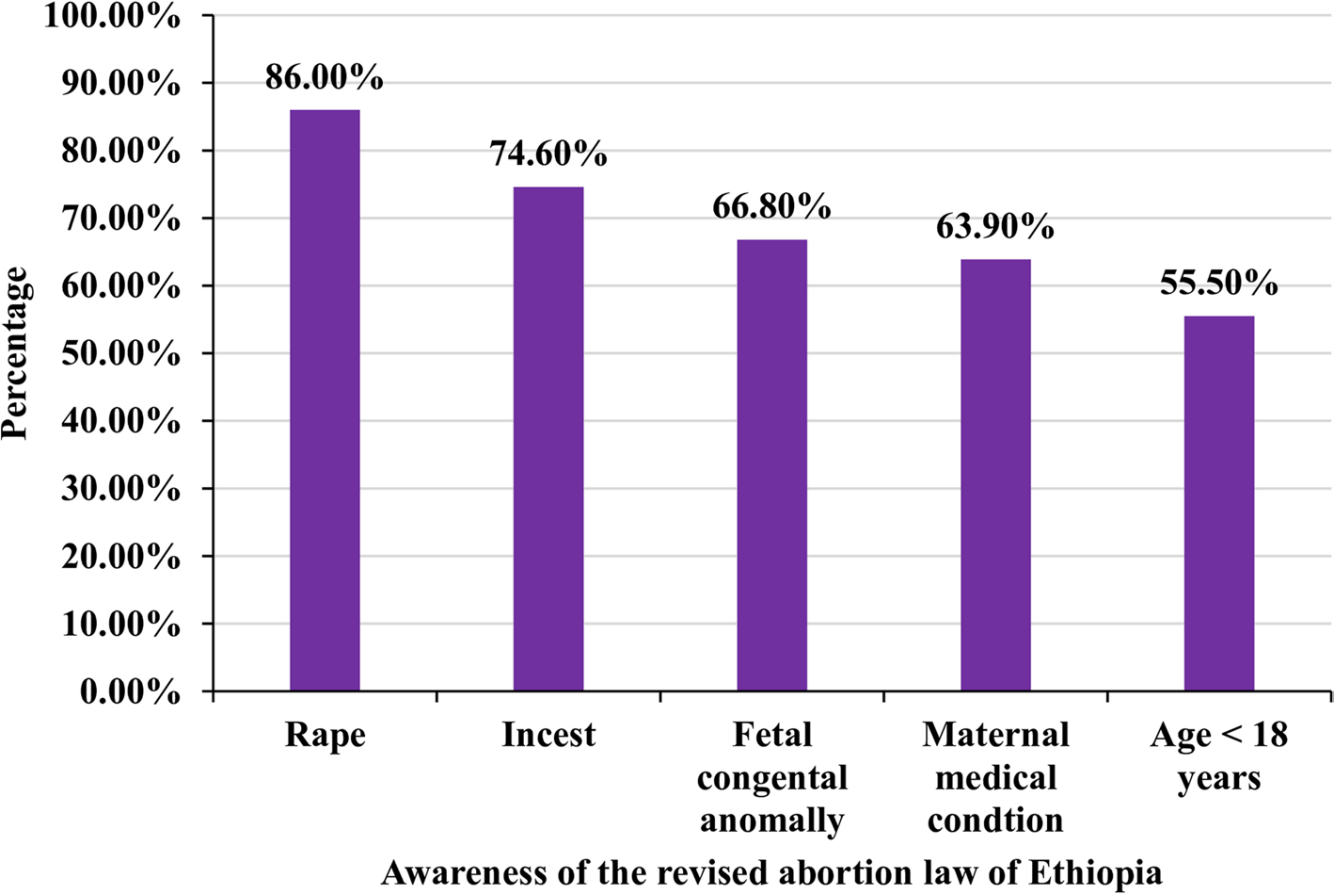
Awareness of the revised abortion law of the country among health care providers in the public health facilities of Bahir Dar city, North-west, Ethiopia, 2021, (*n* = 413)

Regarding the revised abortion law of the country, 355 (86.0%) of the health care providers responded that rape is one of the revised laws, and overall, 301 (72.9%) had a good awareness of the revised abortion law of the country (Fig. 1).

### The practice of safe abortion procedure

Less than half, 173 (41.9%) of the health care providers had trained on abortion, and 145 (35.1%) practiced abortion procedures. Among health care providers who have practiced abortion procedures 78 (53.8%) were currently performing the safe abortion procedure and 115 (79%) performed manual vacuum aspiration (Table 3).

**Table 3:** Practice of safe abortion procedure among health care providers at the public health facilities in Bahir Dar City, Northwest, Ethiopia, 2021, (*n* = 413)

Variables	No. (%)
Trained on safe abortion	
No	240 (58.1)
Yes	173 (41.9)
Ever practiced a safe abortion procedure	
No	268 (64.9)
Yes	145 (35.1)
If you are practicing, when did you perform (*n* = 145)	
I am currently working	78 (53.8)
In the last six month	22 (15.2)
In the last two years	22 (15.2)
More than two years	23 (15.9)
Types of procedure performed (*n* = 145)	
Manual vacuum aspiration	115 (79.3)
Medication abortion	110 (75.9)
Oxytocin induction	41 (28.3)
Dilatation and Evacuation	28 (19.3)

### Reasons for not practicing safe abortion procedures

**Fig. 2:**
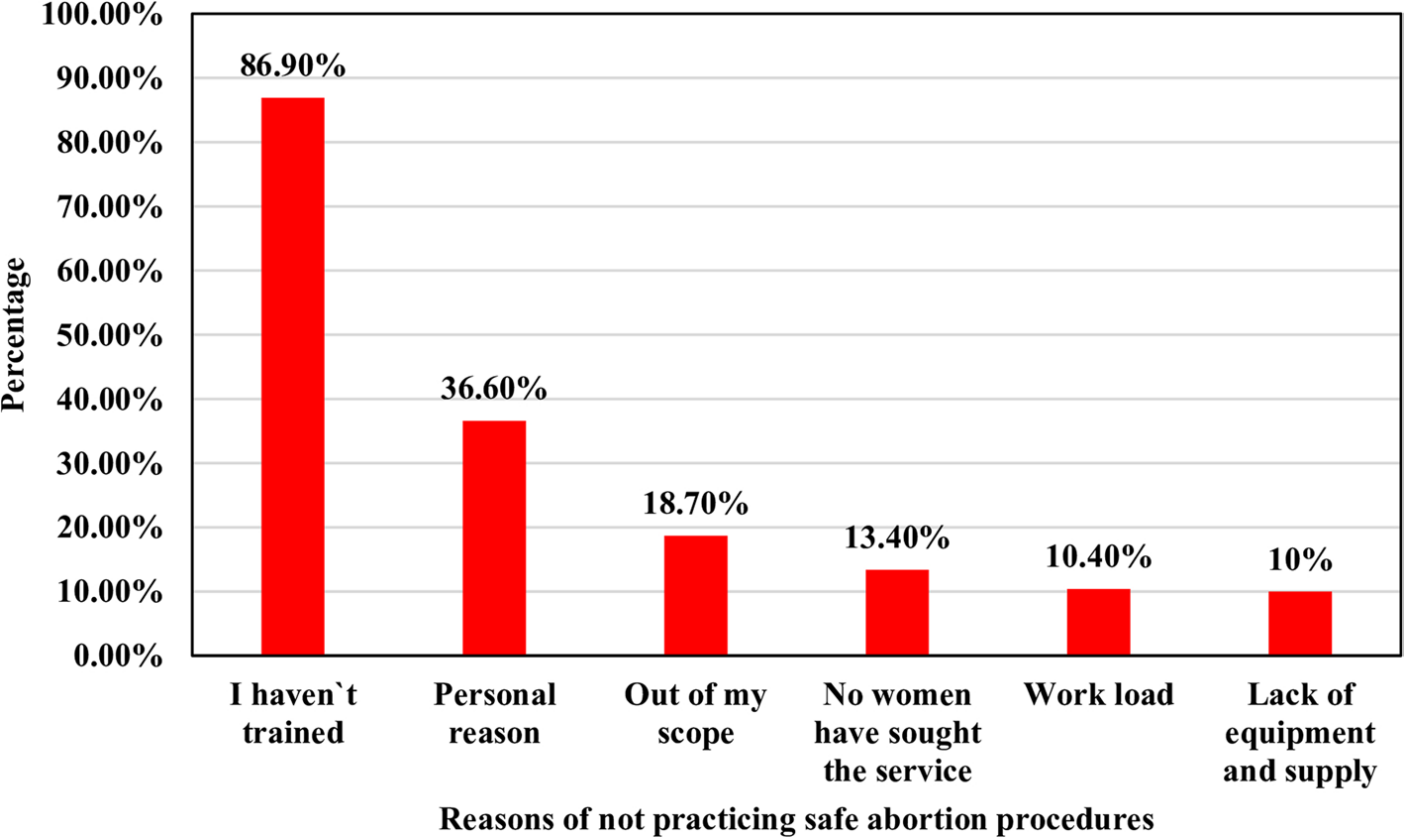
Reason for not practicing/performing abortion procedures among health care providers in the public health facilities of Bahir Dar city, North-west, Ethiopia, 2021, (*n* = 268)

Among health care providers who have not performed the abortion procedure, lack of training on abortion technique was responded by 233 (86.9%) of participants as a major reason for not practicing abortion procedure and followed by personal reason 98 (36.6%) (Fig. 2).

### The attitude of healthcare providers toward safe abortion

Overall, in this study, 290 (70.2%) of the health care providers had a favorable attitude towards safe abortion care. Nearly 33.0% of the health care providers agreed that working in a facility/unit where termination of pregnancy is performed gives comfort. About, 185 (44.8%), 181 (43.8%), 219 (53.0%), 166 (40.2%), 149 (36.1%), and 202 (48.9%) of the health care providers strongly agreed that safe abortion should be performed for raped women, pregnancy because of incest if the pregnancy endangers the health or life of the woman, women with mental disabilities, age under 18 years and in cases of fatal congenital anomaly respectively. From the total health care provider, 152 (36.8%) agreed that abortion services should be accessible under any circumstance and 159 (38.5%) agreed that abortion should be legalized further. Of the health care providers 159 (38.5%) agreed that all health care providers should be able to provide medical abortion for first-trimester pregnancy and 148 (35.8%) disagreed that all health providers should be able to provide surgical abortion for first-trimester pregnancy (Table 4).

**Table 4:** Attitude of health care providers towards safe abortion care at public health facilities in Bahir Dar City, Northwest, Ethiopia, 2021, (*n* = 413)

Variables	Strongly agree	Agree	Neutral	Disagree	Strongly disagree
Having inadequate knowledge of abortion makes the woman abort	38.3%	36.3%	4.1%	14.3%	7.0%
The economic constraint is one of the reasons that women abort	41.9%	40.7%	4.6%	10.4%	2.4%
Women used abortion as a contraceptive method	30.5%	29.8%	9.9%	16.2%	13.6%
Women abort the fetus to avoid unwanted pregnancy	41.6%	40.0%	5.6%	10.2%	2.7%
Women seek abortion services for health reasons	38.3%	37.5%	5.1%	14.0%	5.1%
Women abort the fetus because of partner pressure	32.2%	32.9%	9.2%	17.2%	8.5%
Not being married is a reason for an abortion	34.1%	34.1%	8.7%	15.7%	7.3%
Working in the abortion unit gives comfort	28.6%	32.7%	14.5%	18.6%	5.6%
Abortion should be performed for raped women	44.8%	39.7%	2.7%	6.3%	6.5%
Abortion should be performed for women who become pregnant because of incest	43.8%	38.5%	5.1%	7.0%	5.6%
Abortion should be performed if the pregnancy endangers the health of the woman	53.0%	38.7%	3.9%	3.4%	1.0%
Abortion should be performed for women with mental disabilities	40.2%	37.8%	6.3%	9.0%	6.8%
Abortion should be performed if she seeks and her age is under 18 years	36.1%	34.4%	7.3%	10.9%	11.4%
Abortion should be performed if the fetus has a congenital anomaly	48.9%	42.4%	3.6%	4.1%	1.0%
Abortion service should be accessible under any circumstance	16.2%	36.8%	10.7%	26.4%	9.9%
Legal/safe abortion should be legalized further	17.2%	38.5%	8.7%	25.7%	9.9%
Abortion should be legal for unplanned pregnancy	7.0%	35.6%	13.1%	31.7%	12.6%
Performing medical abortion is more comfortable than surgical abortion	11.4%	49.4%	12.1%	18.9%	8.2%
Medical abortion during 1^st^ trimester of pregnancy should be performed by all healthcare provider	14.5%	38.5%	5.8%	32.2%	9.0%
Surgical abortion in 1^st^ trimester of pregnancy should be performed by all healthcare providers	1.0%	26.2%	2.4%	35.8%	34.6%
Attitude toward safe abortion					
Favorable attitude	290 (70.20)				
Unfavorable attitude	123 (29.80)				

### Reason for the need for further legalization of abortion law

**Fig. 3:**
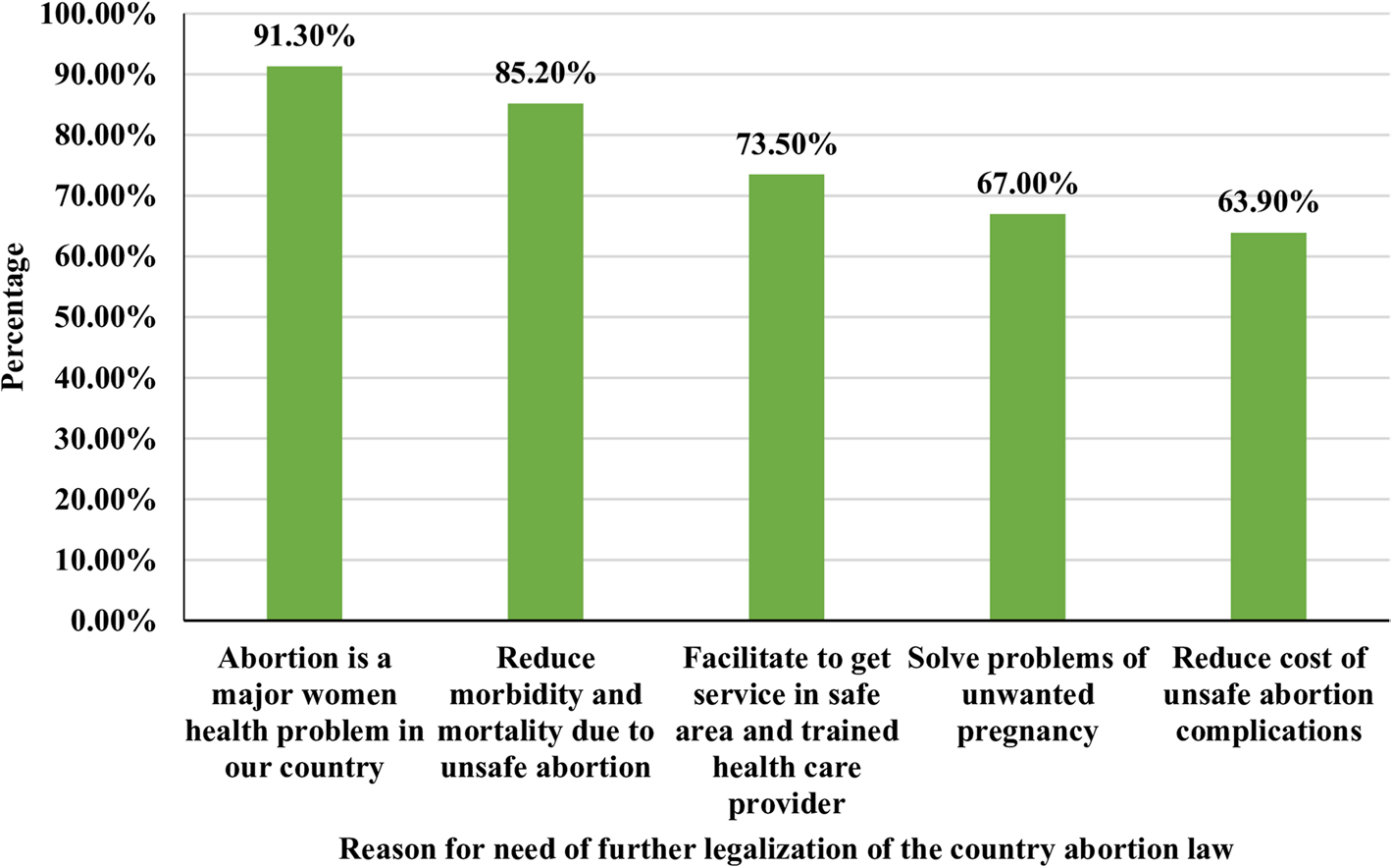
Reason for the need for further legalization of the country abortion law among health care providers in the public health facilities of Bahir Dar city, North-west, Ethiopia, 2021, (*n* = 230)

In the present study, 230 (55.7%) healthcare providers need further legalization of the country's abortion law and among them, 210 (91.3%) responded abortion is the major women's health problem in the country as their main reason (Fig. 3).

### Reason for no need for further legalization of abortion law

**Fig. 4:**
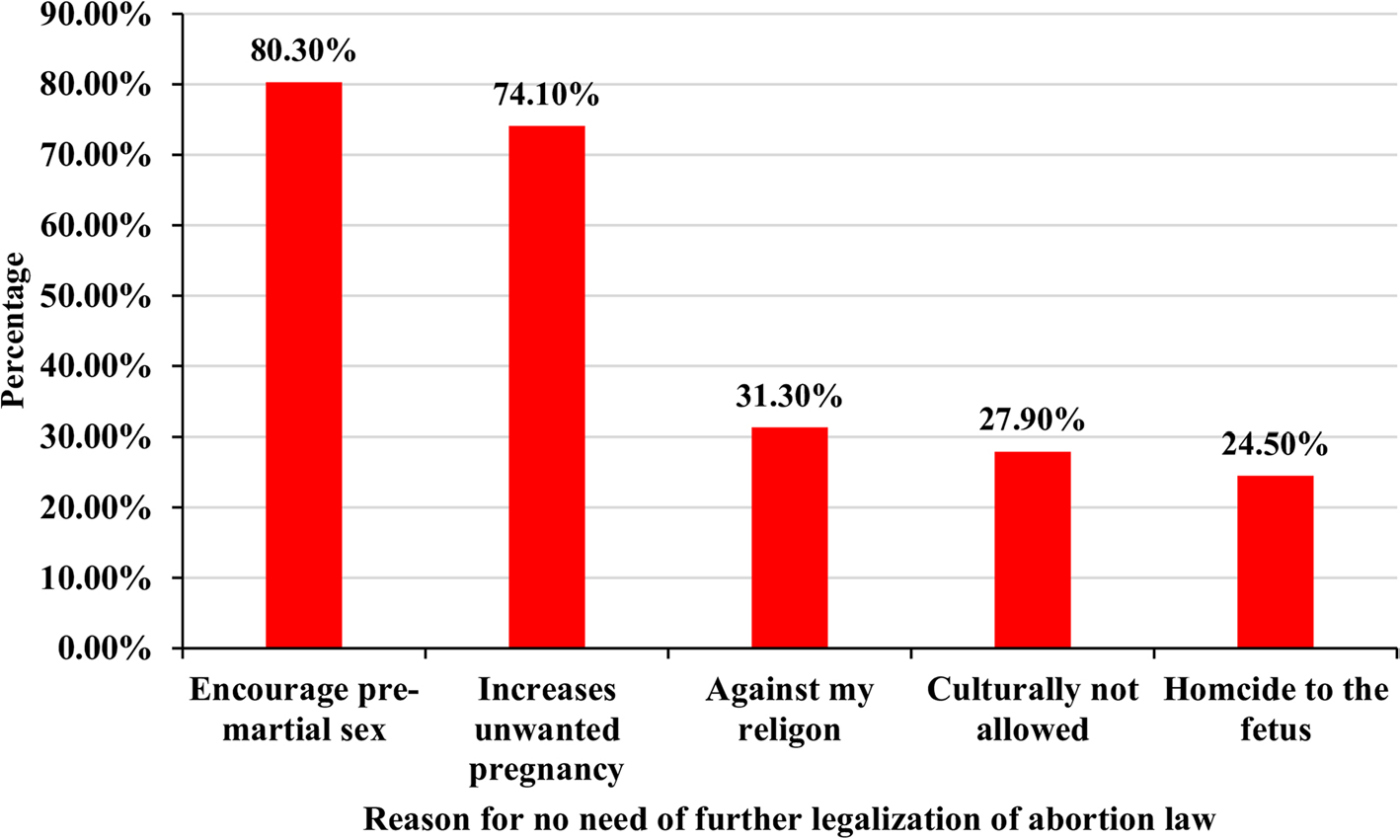
Reason for no need for further legalization of the country abortion law among health care providers in the public health facilities of Bahir Dar city, North-west, Ethiopia, 2021, (*n* = 147)

About one-third (*n* = 147, 35.6%) of the health care providers said that no need for further legalization of the country's abortion law, and their main reason was it may encourage pre/extramarital sex 118 (80.30%), and increase the numbers of unwanted pregnancy 109 (74.2%) (Fig. 4).

### Factors associated with the attitude of health care providers towards safe abortion care

In bivariate analysis: age, sex, profession, working facility, work experience, working unit, training on abortion, practicing of an abortion procedure, awareness of revised abortion law of the country, need for further legalization of the country abortion law, and knowledge of abortion was significantly associated with the favorable attitude of the health care providers towards safe abortion care at a *P*-value of < 0.25.

In a multivariable analysis health care providers who are found in the age group of 25–29, 30–34, and greater than or equal to 35 years [AOR = 3.34, 95% CI = 1.03–10.85], 4.58 [AOR = 4.58, 95% CI = 1.33- 15.83], and 5.30 [AOR = 5.30, 95% CI = 1.43–19.66] respectively, male health care providers [AOR = 3.20, 95% CI = 1.55–6.60], midwives health professionals [AOR = 6.50, 95% CI = 2.40–17.44], ever trained on safe abortion [AOR = 5.09, 95% CI = 2.29–11.32], practicing abortion procedures [AOR = 2.52, 95%, CI = 1.13–5.60], health care provider who is working at a hospital [AOR = 4.77, 95% CI = 1.53–14.91], having good knowledge of abortion [AOR = 7.35, 95% CI = 3.23–16.71], having a good awareness of the revised abortion law of the country [AOR = 6.44, 95% CI = 3.15–13.17] and need further legalization of abortion law [AOR = 11.78, 95% CI = 5.52–24.26] were significantly associated with a favorable attitude of the health care providers towards safe abortion care at a P-value of less than 0.05 (Table 5).

**Table 5:** Logistic regression analysis for the attitude towards safe abortion care among health care providers at the public health facilities in Bahir Dar City, Northwest, Ethiopia, 2021, (*n* = 413)

**Variables**	**Attitude toward safe abortion care**		**COR**^**b**^** (95%-CI)**	**AOR**^**b**^** (95%-CI)**	***P*-value**
	**Favorable**	**Unfavorable**			
Healthcare providers age in years					
20–24	9	26	1	1	
25–29	107	51	6.06 (2.65–13.87)	**3.34 (1.03–10.85)**	0.045*
30–34	110	28	11.35 (4.78–26.93)	**4.58 (1.33–15.83)**	0.016*
≥ 35	64	18	10.27 (4.09–25.80)	**5.30 (1.43–19.66)**	0.013*
Sex of healthcare providers					
Female	135	86	1	1	
Male	155	37	2.67 (1.70–4.18)	**3.20 (1.55–6.60)**	0.002*
Profession					
Nurse	118	83	1	1	
Midwives	99	12	5.80 (2.99–11.25)	**6.47 (2.40–17.44)**	0.001*
Health officer	14	14	0.70 (0.32–1.55)	0.57 (0.17–1.99)	0.381
GP/Specialist/IESO	59	14	2.96 (1.55–5.66)	1.60 (0.60–4.25)	0.345
Working unit					
Outpatient department	46	49	1	1	
Medical ward	37	17	2.32 (1.15–6.67)	2.33 (0.80-.6.79)	0.122
Surgical ward	24	8	3.20 (1.31–7.83)	2.55 (0.60–10.79)	0.202
Pediatrics ward	20	6	3.55 (1.31–9.62)	3.24 (0.74–14.23)	0.199
Maternal and child health	92	14	7.00 (3.51–13.98)	3.28 (0.72–14.98)	0.125
Gynecology ward	38	77	5.78 (2.35–14.24)	3.78 (0.96–14.85)	0.570
Others^a^	33	22	1.60 (0.82–3.13)	2.31 (0.74–7.19)	0.150
Work experience					
Less than 1 year	8	6	1	1	
1–5 years	74	45	1.23 (0.40–3.79)	2.04 (0.32–12.93)	0.451
6–10 years	149	55	2.03 (0.67–6.12)	1.49 (0.25–8.77)	0.662
More than 10 years	59	17	2.60 (0.79–8.54)	1.23 (0.18–8.28)	0.829
Working facility					
Health center	19	23	1	1	
Hospital	271	100	3.28 (1.71–6.28)	**4.77 (1.53–14.91)**	0.007*
Ever trained on safe abortion					
No	136	104	1	1	
Yes	154	19	6.20 (3.61–10.64)	**5.09 (2.29–11.32)**	0.001*
Practiced abortion procedure					
No	169	99	1	1	
Yes	121	24	2.95 (1.79–4.89)	**2.52 (1.13–5.60)**	0.042*
Awareness of revised law					
Poor awareness	42	70	1	1	
Good awareness	248	53	7.80 (4.81–12.66)	**6.44 (3.15–13.17)**	0.001*
Need further legalization of abortion law					
No	87	96	1	1	
Yes	203	27	8.30 (5.05–13.62)	**11.78 (5.72–24.26)**	0.001*
Knowledge of abortion					
Poor knowledge	19	40	1	1	
Good knowledge	271	83	6.87 (3.78–12.51)	**7.35 (3.23–16.71)**	0.001*

^*^Significant at a *P*-value of < 0.05, ^a^Neonatal intensive care unit, Emergency, Operation room, and Recovery unit

^b^*COR*  Crude Odds Ratio, *AOR* Adjusted Odds Ratio, *CI* Confidence Interval

## Discussion

The Sustainable Development Goals (SDG) target 3.1 intended to reduce maternal mortality (MMR) below 70 deaths per 100,000 live births and target 5.6 to ensure universal access to sexual and reproductive health and reproductive rights by the year 2030 [40]. To achieve these goals healthcare providers have a crucial role, as healthcare providers' attitudes play a key role in the care and prevention of unsafe abortion and treatment of abortion-related complications [41]. Health care providers who have a favorable attitude can provide rights-based, fearless, non-judgmental care, and have a non-discriminatory approach toward women [42]. The current study shows that 70.2% [95% CI: 65.6–74.6] of the health care providers had a favorable attitude towards safe abortion care. It is in line with a study done in Asella (68.1%) [43].

However, this finding is higher than the studies conducted in public health facilities of Harar city (58.4%) [44], East Gojjam (56.7%) [36], Addis Ababa health centers (54.1%) [45], Addis Ababa health facilities (51.8%) [21], and Adama health facilities (48.1%) [30]. This discrepancy might be attributed to the differences in the healthcare management of the health facilities. Healthcare facilities that facilitate refreshment training for their healthcare providers could have a well-behaved healthcare provider. Through time different stakeholders give training on safe abortion, thus might be another reason for the improvement of the healthcare provider's attitude toward safe abortion care. Similarly, it is also higher than studies conducted in Zambia (21.4%) [33], Uganda 48% [26], Jamaica (64%) [46], Bengal, India (61.9%) [47], India (40%) [48], and Iran 13.1% [49]. The possible reason might be the difference in the place of residence, the socio-demographic characteristics of the study population, and the health care management systems of the countries.

However, the attitude of health care providers towards safe abortion care is lower than a study conducted in Mekelle shows that 95% of respondents have a favorable attitude towards safe abortion care [50]. The possible reason for this discrepancy might be the working area of the healthcare providers. The study done in Mekelle included only healthcare providers who are working in the hospital, while our study included healthcare providers who are working in hospitals and health centers. As seen in our study, among healthcare providers who are working in the hospital, 73% have a favorable attitude towards safe abortion and only 45.0% of the healthcare providers working in health centers had a favorable attitude.

In this study, socio-demographic characteristics, knowledge, and practice-related factors were significantly associated with a favorable attitude of healthcare providers toward safe abortion care. Health care providers who are found in the age group of 25–29, 30–34, and greater than or equal to 35 years were 3.34, 4.58, and 5.30 times more likely to have a favorable attitude toward safe abortion care respectively. This finding is in line with studies conducted in Ethiopia [30, 36]. This finding of this study is also supported by studies conducted in Nigeria and Ghana [51], Zimbabwe, Uganda, Zambia, and Chile [26, 32, 33, 43]. The possible reason might be those who are found in the age group above 25 years may have more work experience and this could increase their chance of developing a favorable attitude towards safe abortion. There is a supporting report from studies conducted in Mekelle, Asella, and Uganda, which show that healthcare providers who had more experience in work had a favorable attitude towards safe abortion care [26, 50, 52].

Male healthcare providers were 3.20 times more likely to have a favorable attitude toward safe abortion. This finding is in line with other studies [43–45]. The possible reason might be, as we know female health care providers are also part of the general population and they may encounter abortion in their life. Because of this, they may have more understanding of abortion and its complications than males, additionally, as a mother, they may want to continue the pregnancy rather than abort it. Thus, all this might make them have a less favorable attitude towards abortion than male healthcare providers. On the other hand, the finding of this study disagrees with the studies conducted in South Africa, Uganda, and Zambia showing that female healthcare providers had a favorable attitude towards safe abortion [26, 33, 53]. The reason might be value clarification and sensitization among female healthcare providers.

Midwives were 6.49 times more likely to have a favorable attitude towards safe abortion care. This finding agrees with another study [45]. This may be due to midwives' daily activity being with the maternal side, which may help them to have a better understanding related to abortion. Working in the hospital increased the odds of having a favorable attitude toward safe abortion by 4.77 times. This finding is in line with a study conducted in east Gojjam [36]. The possible reason for this might be health care providers who are working in hospitals could have a higher chance of getting adequate and functional equipment for abortion procedures relative to those who are working in health centers. There is a supporting report from studies conducted in Adama and Mekelle shows that the presence of adequate and functional equipment in the facility is positively associated with the attitude of healthcare providers toward safe abortion care [30, 50].

Healthcare providers who are ever trained on abortion were increasing the odds of having a favorable attitude towards safe abortion care by 5.09 times. This finding is congruent with other studies [36, 44]. The possible reason might be that trained personnel may be more aware of the benefits and revised legal abortion laws of the country than those who are not trained. Also, because of having the training they are maybe certified and value verified, thus increasing their chance of having a favorable attitude towards safe abortion care. Practicing abortion procedures increased the odds of having a favorable attitude towards safe abortion care by 2.52 times. This finding was supported by another study [21]. The possible reason for this might be that healthcare providers who practiced/performed abortion procedures may have a high chance of getting information about abortion and it may make them have a favorable attitude towards safe abortion care.

Healthcare providers who had a good awareness of the revised abortion law of Ethiopia were 6.44 times more likely to have a favorable attitude toward safe abortion care. This finding is supported by other studies conducted in Ethiopia, which show that healthcare providers who know the law governing abortion are more likely to have a favorable attitude than those who lack this awareness [21, 44]. Those who need further legalization of abortion law were 11.78 times more likely to have a favorable attitude towards safe abortion care. It is consistent with other studies [30, 36, 44]. The reason may be those health care providers who know the present revised abortion law have a positive outcome and because of this, they may want to have an additional law that will strengthen the present law.

Healthcare providers who had good knowledge of safe abortion were 7.35 times more likely to have a favorable attitude towards safe abortion care. This finding is consistent with studies conducted in East Gojjam [36], Asella [52], South Africa [54], and Nepal [55]. Healthcare providers who have good knowledge of abortion might know the benefits of performing safe abortion procedures for the health of the women as well as for the general population.

### Limitations of the study

Since, the study population were health care providers, because of this there could be a social desirability bias which may have influenced them to identify a more favorable attitude regarding reproductive rights. Another limitation of this study was the exclusion of private clinic health care providers and it was not triangulated with a qualitative study.

### Conclusion and recommendations

In our study, health care providers who had a favorable attitude towards safe abortion care were higher compared to the majority of the studies conducted in different parts of Ethiopia. Age greater than 25 years, male sex, midwife profession, working at the hospital, having abortion training, practicing abortion procedure, having a good awareness of the revised abortion law of the country, need of further legalization of the country abortion law and having good knowledge of abortion were predictors of favorable attitude towards safe abortion care. Access to training and further opportunities for health care providers to attend onsite values clarification workshops and safe abortion training needs to be encouraged and strengthened. It is better to consider the need for sensitization for health care professionals about safe abortion and to create a safe/comfortable environment to perform the safe abortion. In general attitude, transformation is a key intervention to help improve the attitude of healthcare providers, especially those working in reproductive healthcare points including termination of pregnancy when medically indicated.

### Supplementary Information

Supplementary Material 1.

## Data Availability

All related data have been presented within the manuscript. The data set supporting the conclusion of this article is available from the corresponding author upon reasonable request. Wondu Feyisa Balcha: (email:wondufeyisaa85@gmail.com). ORCID iDs: http://orcid.org/0000-0001-7639-3363
