# Supplementary material for: Health care providers attitude towards safe abortion care and its associated factors in Northwest, Ethiopia, 2021: a health facility-based cross-sectional study
Source: Reprod Health. 2024 Jun 8;21:83. doi: 10.1186/s12978-024-01826-w (PMC11162021; doi:10.1186/s12978-024-01826-w)
Supplement: Supplementary file 1 — Supplementary Material 1. [file 12978_2024_1826_MOESM1_ESM.pdf]

ANNEXES

Annex I: Information Sheet and Informed Consent

Information sheet

Title: attitude towards safe abortion and associated factors among health care providers at public health facilities of Bahir Dar city, Northwest Ethiopia, 2021.

Project owner Organization: Bahir Dar University College of medicine and health sciences school of public health.

Sponsored organization: self-sponsored

Purpose: The aim of the study is to assess the attitude towards safe abortion and associated factors among healthcare providers at public health facilities in Bahir Dar city, Ethiopia.

Risk and benefit: There is no risk in participating in this research project and if you participate in this study, there may not be direct benefit to you; but, your participation is very important to conduct this study. Ultimately, this will help us to identify the gap and take appropriate measures by concerned bodies. So, you are requested to spend your time willingly to respond your information to the questionnaire.

Confidentiality: The information collected from this study will be kept confidential and the information about you will be collected for this study will be stored in a file without your name, but a code number will be given to it. In addition, it will not be revealed to anyone except the principal investigator and will be kept locked with key.

Right to refusal: You have the right to refuse from participating in this study. You can choose not to respond to some or all questions if you do not want to give your response. You have also a full right to withdraw from this study at any time whenever you want, and this will not label you for any loss of benefits which you otherwise are entitled. You have the full right not to answer any question that you do not want to answer it.

Duration of interview: The interview of all questions will be taken 15-20 minute

Consent form

How are you? I am_____________, I would like to ask you few questions and your willingness in the study is essential. It is a self-administrative questionnaire.

This study is prepared to obtain relevant information about "**Attitude towards safe abortion and associated factors among health care providers at public health facilities of Bahir Dar city Northwest Ethiopia, 2021".** Your participation in the study is very important in reducing the maternal morbidity and mortality rate, which caused by unsafe abortion and its complication.

Your name & address will not be written in this form and will never be used in connection with any information you tell us. All the information given by you will be kept strictly confidential and only used for this study. Your participation is voluntary and you are not obligated to answer any question which you do not wish to answer. If you fill discomfort to respond to any of the question, please fill free to drop it any time you wish to do so.

I have read all the process and the objective of the study and I have understood the same as written. I understood that the research imposes no risk would be provided to me and families.

Could I have your permission to continue?

1. Yes ☐ 2. No ☐

The principal Investigator address: phone No. +251-920771673

E-mail= abebaytadie2004@gmail.com

Date of Data Collection: __________ Name of Health Facility ________________

Sub-city ____________ Health Facility Code ______________

Result: Questionnaire completed _________________________

Questionnaire partially completed ________________

Checked by Supervisor: Name _____________________________________

Supervisor's Signature _______________________ Date _______________

Annex II: English Version questionnaires

Bahir Dar University, College of medicine and Health Sciences, School of Public Health

Department of Reproductive Health & Population studies

This questionnaire is adapted and modified from different related researches that were done previously**.**

Questionnaire code --------------------

Instruction:-write the correct response based on the requirement of the questioner.

Part I: Socio-demographic characteristics of the health care providers

S. No	Question	Response	Skip
101	Age	________years	
102	Sex	1. Female 2. Male	
103	Marital status	1. Single 3. Divorced<br>2. Married 4. Windowed	
104	Religion	1. Orthodox 3. Muslim<br>2. Protestant 4. Catholic	
105	Profession	1. Midwife 4. HO<br>2. Nurse 5. GP<br>3. Specialist (Obstetrician) 6. IESO	
106	Working facility	1. Hospital 2. Health center	
107	Working unit	1. MCH<br>2. OPD<br>3. Medical ward<br>4. Surgical ward<br>5. Pediatric ward<br>6. Gynecology ward<br>7. Operation room<br>8. Recovery<br>9. Emergency<br>10. NICU	
108	Work experience	------------years	

Part II: Knowledge related question on safe abortion

S.no	Variable	Response	Skip
201	Abortion in Ethiopia context	1. Termination of pregnancy before 20 weeks of GA from LNMP<br>2. Termination of pregnancy before 28 weeks of GA from LNMP	
202	Best time for safe abortion	1. After 12 weeks of GA<br>2. Before 12 weeks OF GA	
203	Types of abortion method you know (more than one answer is possible)	1. Manual vacuum aspiration (MVA) (Yes/no)	

		2. Medical abortion (using mifepristone and misoprostol)) (Yes/no)<br>3. Dilatation and evacuation) (Yes/no)<br>4. Oxytocin induction (Yes/no)	
204	Revised abortion law of Ethiopia (more than one answer is possible)	1. Rape (Yes/no)<br>2. Incest (Yes/no)<br>3. Age younger than 18 years old (Yes/no)<br>4. Maternal medical condition) (Yes/no)<br>5. Foetal congenital anomaly) (Yes/no)	
205	Place for terminating pregnancy as permitted by the revised abortion law of Ethiopia	1. Non-equipped health facilities that are not authorized to perform the procedure with no trained staffs<br>2. Equipped health facilities with trained staffs that are authorized to perform the procedure with trained staffs	
206	Raped women should submit evidence of rape in order to obtain abortion service	1. Yes<br>2. No	
207	The women who requested termination of pregnancy due to incest should submit evidence	1. Yes<br>2. No	
208	The health care provider has to secure on informed consent using a standard consent form	1. Yes<br>2. No	
209	Health care provider has an ethical obligation not to disclose the information provided by the woman unless permitted by the woman or ordered by the court	1. Yes<br>2. No	
210	Components of Post abortion care (PAC) (more than one answer is possible)	1. Community and service provider partnership<br>2. Counselling<br>3. Treatment of incomplete and complication of unsafe abortion<br>4. Contraceptive and FP service provision<br>5. Integration to RH and other Service	
211	Who do you think can perform MVA according to FMoH guideline? (more than one answer is possible)	1. Specialists (Obstetricians) 4. HO<br>2. IESO 5. Midwives<br>3. GPs 6.Nurses	
212	Who do you think can perform medication abortion according to FMoH guideline? (more than one answer is possible)	1. Specialists (Obstetricians) 4. HO<br>2. IESO 5. Midwives<br>3. GPs 6. Nurses	

213	Who do you think can perform dilatation and evacuation according to FMoH guideline? (more than one answer is possible)	1. Specialists (Obstetricians) 4. HO<br>2. IESO 5. Midwives<br>3. GPs 6. Nurses	
214	Who do you think authorized to perform 2nd trimester abortion procedures (more than one answer is possible)	1. Specialists (obstetricians) 4. HO<br>2. IESO 5. Midwives<br>3. GPs 6. Nurses	
215	Referral arrangement for social support and care is an integral part of overall abortion care	1. Yes<br>2. No	
216	HC expected to give 1st trimester safe abortion services	1. Yes<br>2. No	
217	Who do you think can provide education on legal provision of abortion (more than one answer is possible)	1. Specialists 4. HO<br>2. IESO 5. Midwives<br>3. GPs 6.Nurses	

Part III: Practice related questions on safe abortion

S.no	Variable	Response	Skip
301	Are you trained on abortion	1. Yes<br>2. No	
302	Have you ever performed/practiced abortion procedure	1. Yes<br>2. No	If 2 skip 303 & 304
303	If you are practicing, when did you perform?	1. I am currently working<br>2. In the last six month<br>3. In the last two years<br>4. More than two years	
304	Types of procedure you performed (more than one answer is possible)	1. MVA<br>2. Medication abortion<br>3. Oxytocin induction<br>4. Dilatation & Evacuation	

305	Reason not practicing or performing abortion procedure (more than one answer is possible)	1. I haven't trained on abortion technique<br>2. Lack of equipment and Supply<br>3. Personal reason<br>4. Work overload<br>5. No women have sought this service<br>6. Outside of the scope of my practice	

Part IV: Attitude towards safe abortion

NB. **1. Strongly Disagree, 2. Disagree, 3. Neutral, 4. Agree, 5. Strongly Agree**

S.no	Variable	Response				
401	What do you think if women seek safe abortion?					
	**1. Strongly Disagree, 2. Disagree, 3. Neutral, 4. Agree, 5. Strongly Agree**					
	Inadequate Knowledge	1	2	3	4	5
	Economical constraint	1	2	3	4	5
	Used as a Contraceptive	1	2	3	4	5
	To avoid unwanted pregnancy	1	2	3	4	5
	Health reasons	1	2	3	4	5
	Partner pressure	1	2.	3	4	5
	Not being married	1	2	3	4	5
402	Working in a site where termination of pregnancy is performed give comfort	1	2	3	4	5
403	In what circumstance safe abortion should be given?					
	Rape	1	2	3	4	5
	Incest	1	2	3	4	5
	Endangers the health of the woman	1	2	3	4	5
	Women with mental disabilities	1	2	3	4	5
	If age under 18 years	1	2	3	4	5
	In cases of fetal congenital anomaly	1	2	3	4	5

404	Safe abortion should be accessible under any circumstance	1	2	3	4	5
405	Legal/safe abortion should be further legalized	1	2	3	4	5
406	Abortion should be legal if the pregnancy was unplanned, and the woman does not want to be pregnant	1	2	3	4	5
407	Medical abortion gives more comfort than surgical abortion for first trimester pregnancy.	1	2	3	4	5
408	All health care providers should be able to provide medical abortion for first trimester pregnancy	1	2	3	4	5
409	All health providers should be able to provide surgical abortion for first trimester pregnancy	1	2	3	4	5
410	If you agree/or strongly agree in question number 405, what is your reason (more than one answer is possible)	1. Facilitate to get service in safe area and trained provider<br>2. Abortion is a major women health problem in our area<br>3. Reduce morbidity and mortality due to unsafe abortion<br>4. Reduce cost due to unsafe abortion complication<br>5. Solve problems of unwanted pregnancy				
411	If disagree/ neutral or strongly disagree in question number 405, what is/are your reason (more than one answer is possible)	1. It encourage per/extramarital sex				

		2. It encourage unwanted pregnancy<br>3. Against my religion<br>4. My culture not allowed<br>5. It is homicide to the fetus

Thank you for giving your time to fill the questioner. If you have any additional comments, you can!
